# Maximum temperature accounts for annual soil CO_2_ efflux in temperate forests of Northern China

**DOI:** 10.1038/srep12142

**Published:** 2015-07-16

**Authors:** Zhiyong Zhou, Meili Xu, Fengfeng Kang, Osbert Jianxin Sun

**Affiliations:** 1 Ministry of Education Key Laboratory for Silviculture and Conservation, Beijing Forestry University, Beijing, 100083, China; 2 The Institute of Forestry and Climate Change Research, Beijing Forestry University, Beijing, 100083, China

## Abstract

It will help understand the representation legality of soil temperature to explore the correlations of soil respiration with variant properties of soil temperature. Soil temperature at 10 cm depth was hourly logged through twelve months. Basing on the measured soil temperature, soil respiration at different temporal scales were calculated using empirical functions for temperate forests. On monthly scale, soil respiration significantly correlated with maximum, minimum, mean and accumulated effective soil temperatures. Annual soil respiration varied from 409 g C m^−2^ in coniferous forest to 570 g C m^−2^ in mixed forest and to 692 g C m^−2^ in broadleaved forest, and was markedly explained by mean soil temperatures of the warmest day, July and summer, separately. These three soil temperatures reflected the maximum values on diurnal, monthly and annual scales. In accordance with their higher temperatures, summer soil respiration accounted for 51% of annual soil respiration across forest types, and broadleaved forest also had higher soil organic carbon content (SOC) and soil microbial biomass carbon content (SMBC), but a lower contribution of SMBC to SOC. This added proof to the findings that maximum soil temperature may accelerate the transformation of SOC to CO_2_-C via stimulating activities of soil microorganisms.

Global surface temperature is predicted to keep increasing by about 4 °C until the end of this century, which will particularly accelerate the carbon cycles of terrestrial ecosystems^1^,^2^. Vice versa, increased CO_2_ concentration of atmosphere will inevitably lead to a higher temperature increase in future scenarios of climate change. This apparently demonstrates a tight correlation between soil CO_2_ efflux and temperature. Moreover, their robust relationship virtually depends on the properties of soil temperature and other biophysical factors under diverse environmental conditions.

The CO_2_ emitted from soil surface (i.e. soil respiration) derives from a composite process, normally classified into autotrophic respiration by the activities of plant root, and heterotrophic respiration via the microbial decomposition of soil organic matter^3^,^4^. Its magnitude is conjointly determined by many biotic and abotic factors, including gross primary production, plant photosynthesis, plant roots, soil microbial biomass and activity, soil organic matter content, soil water content, and soil temperature, etc^3^^,^^4^^,^^5^. Furthermore, the interactions among these environmental variables deepen the complexity of the mechanism underlying soil CO_2_ efflux. For example, soil respiration was decoupled from soil temperature when soil moisture content was below the threshold value^6^. In addition, precipitation pulse events could abruptly increase soil respiration rate in short period^7^. It is indicated that empirical models integrating more variables could provide a more accurate estimation of soil CO_2_ efflux than that with a single ecological variable^4^,^8^. Although the effects of other variables on soil respiration have received much attention, soil temperature and soil moisture content are still the two predominant variables extensively used to explain the variance of soil respiration. In principle, temperature is the most fundamental parameter, around which many models have been built, including the well-known Arrhenius function^9^. The effects of other environmental factors could be reduced by modifying the empirical models through clarifying the magnitude of the temperature dependence of soil repiration.

Accumulative soil CO_2_ efflux on a long-term scale is a vital component of ecosystem respiration, and in turn determines the carbon balance between terrestrial ecosystems and the atmosphere. The emergence of automatically continuous measurement system of soil respiration offers an opportunity to evaluate this carbon balance^7^, however, annual soil respiration is still calculated via empirical functions using continuously (i.e., hourly or daily) monitored data of soil temperature^10^. This is because soil respiration is a temperature dependent process, and these two time series can fit well a number of positive mathematical relationships^2^,^11^. Yet, these empirical functions have been founded basing on the steady state assumption of the temporal correlation between soil respiration and temperature with time. In essence, temporal variation is an inherent property of soil temperature. Although it has been extensively accepted that the seasonal variation of soil respiration is induced by seasonal changes in soil temperature, no much attention has been paid on the effects of maximum or minimum soil temperature on soil CO_2_ efflux.

Soil temperature normally exhibits great heterogeneity and fluctuating values over different periods from hourly to diurnal, seasonal, and annual, especially in temperate regions, which mainly accounts for the temporal variations of soil respiration^7^. This is the reason that long-term soil respiration can be upscale estimated using daily, monthly, and mean annual temperature (MAT)^12^. However, the surrogate efficacy of average soil temperature has been questioned by a number of studies^2^,^4^. First of all, the temporal correlation of soil respiration with temperature has not been found to be significant on every day of the year^4^. Additionally, Bond-Lamberty and Thomson^2^ have demonstrated the vital importance of temperature anomaly in determining the amplitude of soil respiration. These indicate that soil temperature is a more flexible biophysical factor and can reflect the changeable characteristics of natural circumstance. Particularly, the role of extreme soil temperature (i.e. maximum and minimum) still remains unclear in regulating soil respiration. In order to save the enormous efforts in measuring the soil CO_2_ efflux at a long time period, it is worthy to make certain what property of soil temperature can be the suitable surrogate of soil respiration for forest ecosystems.

As the climate has been undergoing a warming scenario, we hypothesized that it was the temperature increase of warm season that would lead to a higher soil CO_2_ efflux of temperate forests. To evaluate the impacts of the warmest soil temperatures at diurnal, monthly and seasonal scales on soil respiration, we measured instantaneous rate of soil respiration and coupled soil temperature periodically, and monitored hourly soil temperature for a whole year at nine forest plots within a catchment of temperate forests in northern China. In this study, our main objectives were to identify (1) the correlation of soil respiration with soil temperatures on variant temporal scales, (2) the manipulation of maximum temperature on accumulative soil CO_2_ efflux, for temperate forests of northern China.

## Results

### Accumulative soil CO_2_ efflux

**Figure 1:**
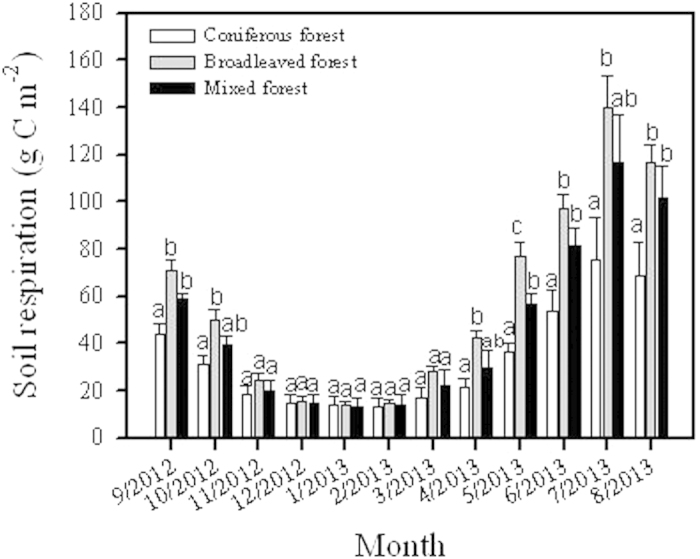
Seasonal variation of soil respiration during September 2012 and August 2013 among three types of temperate forest. Different lowercase letters above bars mean significant difference (*P* < 0.05, n = 3) among these three forest types.

Annual soil respiration markedly varied from 409 g C m^−2^ in coniferous forest to 570 g C m^−2^ in mixed forest and to 692 g C m^−2^ in broadleaved forest (*P* = 0.004, n = 3). Significant difference in monthly soil respiration was also detected among these three forest types through warm seasons from September to October in 2012 and from April to August in 2013. The highest monthly soil respiration turned out in July and was 75.24 g C m^−2^, 139.69 g C m^−2^, and 116.64 g C m^−2^, and the soil respiration in January was lowest at 14.03 g C m^−2^, 13.77 g C m^−2^, and 13.32 g C m^−2^, respectively in coniferous, broadleaved, and mixed forests. Generally, soil CO_2_ efflux during summer (Jun, Jul, Aug) accounted for 50.73% of annual soil respiration for temperate forests. Winter (Dec, Jan, Feb) soil respiration was only about 8.12% of total annual soil CO_2_ efflux. Soil CO_2_ efflux in spring (Mar, Apr, May) and autumn (Sep, Oct, Nov) equally took over 20% or so of the total CO_2_ efflux during the study year (Fig. 1).

### The effects of different forest types on ecological variables

Forest type greatly influenced basal soil respiration (R_10_), SOC, soil total nitrogen content (TN), soil pH, and litter carbon stock in northern China. R_10_ was significantly higher in broadleaved and mixed forests than in coniferous forest (*P* = 0.015, n = 3). Additionally, broadleaved forest also had the highest SOC and TN contents among these three forest types. SOC and TN were 83.62% and 93.10%, 94.82% and 90.48% higher in broadleaved forest than in coniferous and mixed forests, separately. Soil pH of broadleaved forest was significant and 13.02% larger than that of mixed forest. However, litter carbon stock was highest in coniferous forest, and was 80.85% and 94.54% higher than in broadleaved and mixed forests. No significant differences were found in C:N ratio, SMBC, mean annual temperature (MAT), and Q_10_ among these three forest types (Table 1).

### Seasonal variation in soil temperature

**Figure 2:**
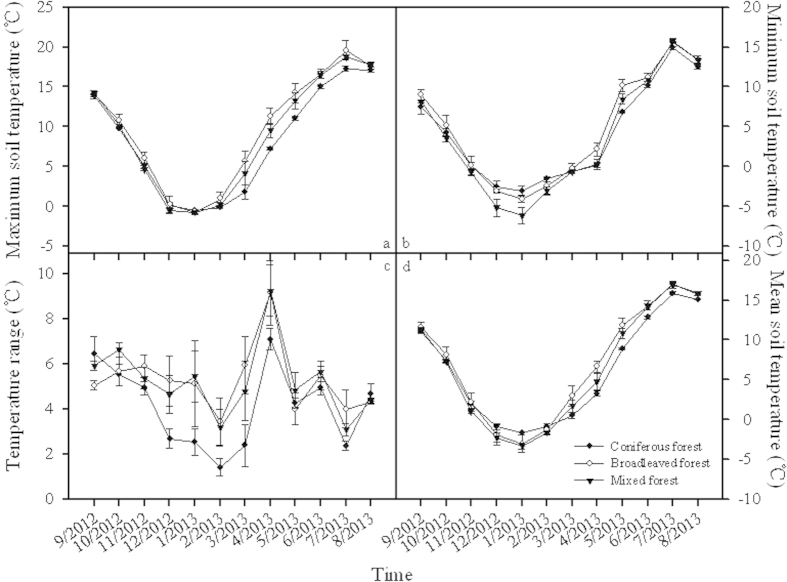
Temporal changes in maximum soil temperature (**a**), minimum soil temperature (**b**), soil temperature range (**c**), and mean soil temperature (**d**) during September 2012 and August 2013 among three types of temperate forest.

Distinct seasonal changes in mean, maximum and minimum soil temperature were detected in these three forest types, with the highest values in July and the lowest values in January, during the study period. On the monthly scale, maximum soil temperature varied from −0.52 °C to 17.27 °C, from −0.72 °C to 19.60 °C, and from −0.73 °C to 18.79 °C, and minimum soil temperature ranged from −3.04 °C to 14.91 °C, from −4.08 °C to 15.61 °C, and from −6.17 °C to 15.71 °C, and mean soil temperature varied from −1.71 °C to 15.89 °C, from −3.03 °C to 16.99 °C, and from −3.32 °C to 16.98 °C, respectively in coniferous forest, broadleaved forest, and mixed forest (Fig. 2a,b,d).

However, there was no general changing trend of soil temperature range among these forest types. The largest value of soil temperature range happened over April, the lowest was over February. The temperature range over the warmest month of July kept the second lowest value (Fig. 2c).

### Correlation of warmest temperature with annual soil respiration

**Figure 3:**
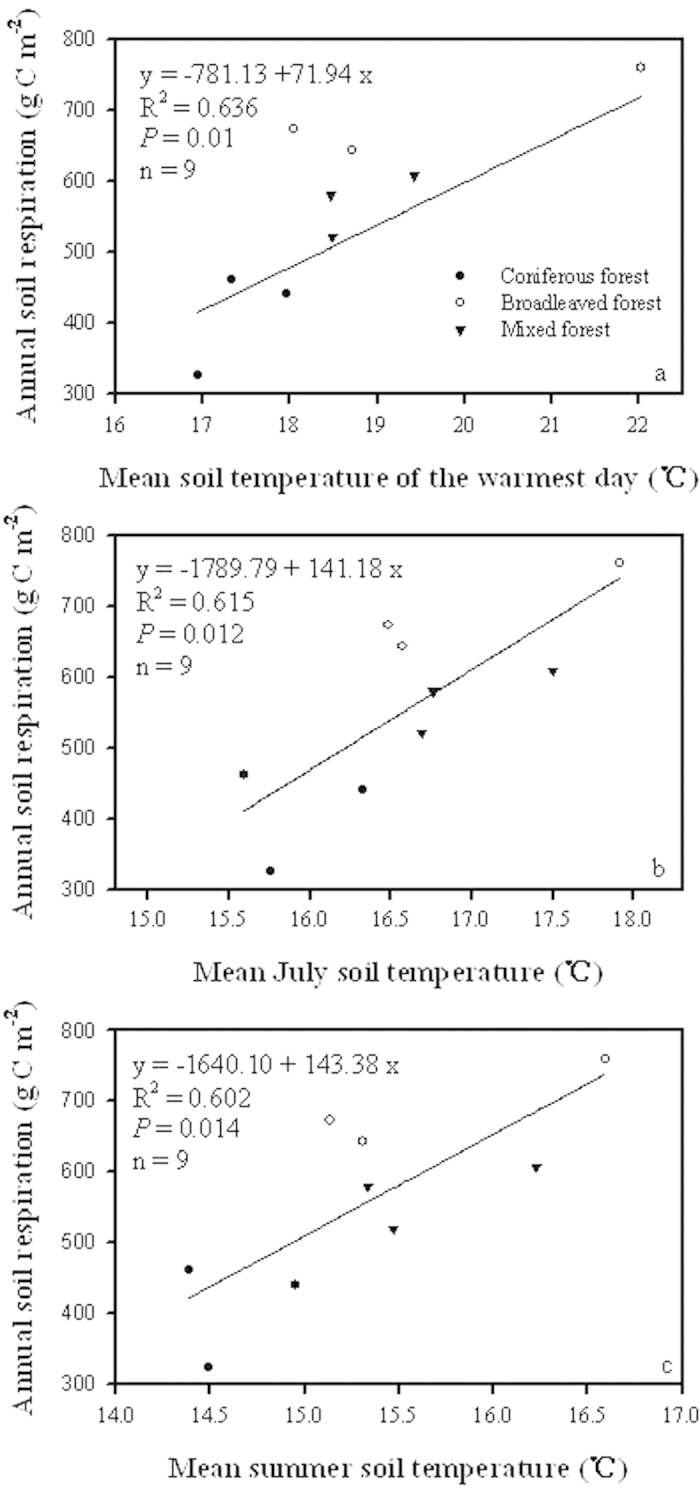
Correlations of soil CO_2_ efflux during September 2012 and August 2013 with mean soil temperature of the warmest day (**a**), mean July soil temperature (**b**), and mean summer soil temperature (**c**) for temperate forests.

In northern China, July and summer are the warmest month and season with highest temperatures. Mean July soil temperature increased from 15.59 °C to 17.92 °C, mean summer soil temperature changed from 14.39 °C to 16.59 °C, and mean soil temperature on the warmest day varied from 16.94 °C to 22.03 °C, during the study period for these three forest types. These three properties of soil temperature extremely affected accumulative soil CO_2_ efflux over an annual scale for temperate forests. Annual soil respiration significantly linearly increased with mean soil temperatures on the warmest day, in July and summer during the study period in temperate forests (Fig. 3a–c). In particular, no significant correlation was found between annual soil CO_2_ efflux and mean annual soil temperature for these three forest types (R2 = 0.43, P = 0. 06, n = 9; original data not shown).

### The variation of soil CO_2_ efflux with soil temperature at monthly scale

**Figure 4:**
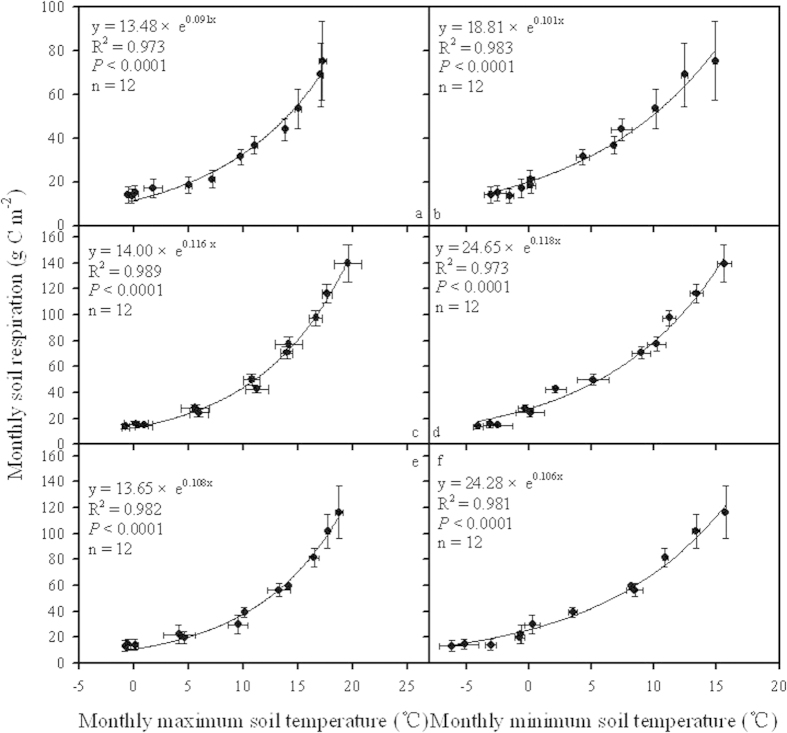
Correlations of monthly soil respiration with maximum and minimum soil temperature during September 2012 and August 2013 for coniferous forest (**a,b**), broadleaved forest (**c,d**) and mixed forest (**e,f**).

**Figure 5:**
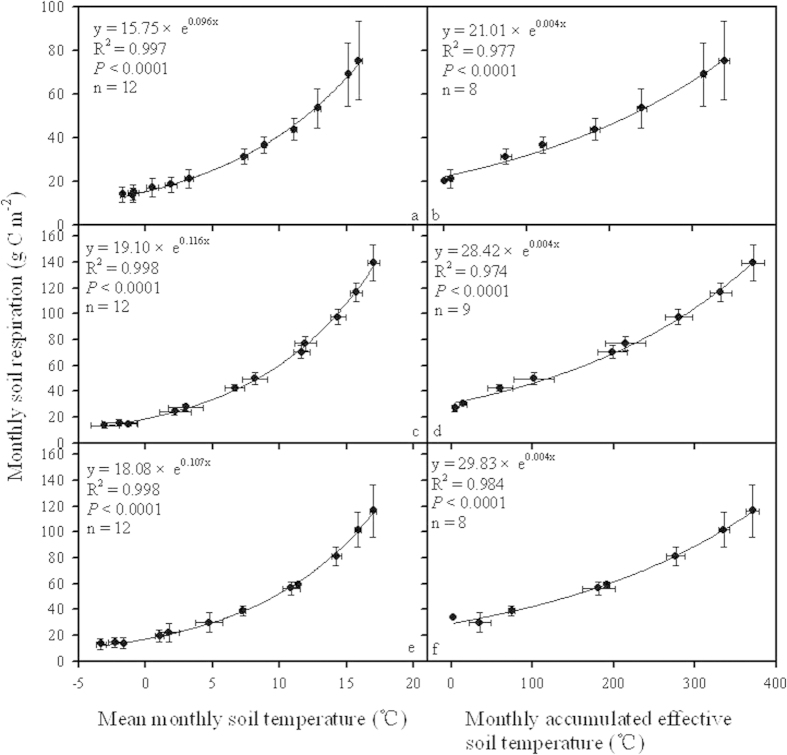
Correlations of monthly soil respiration with mean monthly soil temperature and monthly accumulated effective soil temperature during September 2012 and August 2013 for coniferous forest (**a,b**), broadleaved forest (**c,d**) and mixed forest (**e,f**).

The changing trend of soil CO_2_ efflux was in a good parallel with that of soil temperature on a monthly scale for temperate forests in northern China. Monthly soil respiration was positively and exponentially correlated with the maximum soil temperature, the minimum soil temperature, and mean soil temperature during the corresponding period for all three types of temperate forest (Fig. 4a–f, 5a,c,e). Concurrently, an extremely significant and positive correlation existed between accumulative soil CO_2_ efflux and accumulated effective soil temperature on a monthly scale in the studied forest communities. Most of the variation in monthly soil respiration could be ascribed to the increase in the accumulated effective soil temperature in all three types of temperate forest (Fig. 5b,d,f).

## Discussion

Soil CO_2_ efflux has received a great deal of attention since the earlier studies^4^,^13^, and its importance has been extensively accepted in determining the role of a forest ecosystem as a carbon sink/source^14^. The carbon sequestration of a forest ecosystem largely depends on the difference between net primary production and heterotrophic soil CO_2_ efflux. The CO_2_ efflux through heterotrophic decomposition constitutes at least 50% of total soil respiration in northern temperate forests^15^.

Generally speaking, the dynamic balance between soil organic carbon input and output was virtually determined by the accumulative soil CO_2_ efflux on a normally annual scale. In the study region of northern China, annual soil respiration varied from 409 g C m^−2^ yr^−1^ in coniferous forest to 692 g C m^−2^ yr^−1^ in broadleaved forest, which was comparable to those of similar forest types in the same climate zone^10^,^16^ and fell right within the range of six temperate forests in northeast China^17^. The larger accumulative soil CO_2_ efflux of broadleaved forest was mainly due to its higher soil temperature over different temporal scales, as temperature increase in the warmest period was indicated by this study results to lead to a greater soil respiration.

Soil respiration of temperate forests displayed distinct seasonal variation with nearly 51% CO_2_ emitted in summer and only 8.00% CO_2_ respired in winter. The proportion of winter soil CO_2_ efflux to total annual soil respiration agreed well with 4.92–7.83% of Wang *et al.*^18^. This was mainly attributed to the robust low value of winter soil temperature and the thickness and duration of snow cover^19^. Although winter temperature was indicated to have a vital impact on plant phenology^20^, we speculated that future increase in winter temperature would have limited impact on annual soil CO_2_ efflux in temperate forests of northern China, because of the lowest proportion of winter soil respiration.

Seasonal changes were clearly observed in maximum soil temperature, minimum soil temperature, mean soil temperature, soil temperature range, and accumulated effective soil temperature during the study period of twelve months for the three types of temperate forest in northern China. Generally, broadleaved forest had a comparatively higher value in these properties of soil temperature. This was because the broadleaved forest mainly consisted of *Q. wutaishanica*, which normally distributed along the south direction slope of the investigated mountain. On the northfacing slope stood the coniferous forest with a lower soil temperature.

In addition, soil CO_2_ efflux on a monthly scale in three forest types was largely and significantly explained by the changes in maximum, minimum, mean, and accumulated effective soil temperatures at the corresponding temporal scales. It was evident that the temporal variation of soil respiration greatly depended on the seasonal changing trend of soil temperature in temperate forests. Furthermore, soil CO_2_ efflux on an annual scale was well interpreted respectively by mean soil temperatures on the warmest day, in the warmest month and season for these three temperate forests. This suggested that soil temperature in the warmest period was the most appropriate predictor in modeling the annual soil respiration in temperate forests of northern China. This finding slightly differed from the results of Bahn *et al.*^12^ and Litton *et al.*^21^, who pointed out that total annual CO_2_ emission could be predicted by the soil respiration rate at mean annual temperature or increase with mean annual temperature. In this study, no significant correlation was ever detected between mean annual soil temperature and annual soil CO_2_ efflux for these three temperate forests. Mean annual value may obscure the effects of soil temperature fluctuations at diurnal or seasonal scales on instantaneous rate of soil respiration, especially in temperate climatic zone. Furthermore, water-limitation was perhaps another contributor to the decoupling between mean annual soil temperature and annual soil respiration in the studied temperate forests^2^.

Overall, the surrogate legality of soil temperature also relied on the period length over which accumulative soil CO_2_ efflux was calculated. For temperate forests in northern China, appropriate predicting factors varied from maximum and minimum soil temperatures, mean soil temperature, and accumulated effective soil temperature for the estimation of monthly soil respiration to mean July soil temperature, mean summer soil temperature, and soil temperature of the warmest day for the calculation of annual soil CO_2_ efflux. In these different properties of soil temperature, only the soil temperature in the warmest periods was a most suitable variable to predict the accumulative soil CO_2_ efflux at both monthly and annual scales.

Soil respiration derived from two major biological processes, autotrophic respiration by the growth and maintenance of plant roots, and heterotrophic respiration via the microbial decomposition of carbohydrates from live roots, litter, and old soil organic matter^3^,^4^. Both of these two fluxes were greatly affected by higher temperature. In warm season, plant has higher physiological activity, e.g. plant growth and photosynthesis, which simultaneously induced a larger proportion of autotrophic respiration^22^; soil microbial activity was also motivated by warm soil temperature, which speeded the turnover rate of soil organic carbon and produced more heterotrophic CO_2_ efflux^23^. In this study, broadleaved forest had a significant higher SOC content than both coniferous and mixed forests (*P* = 0.026, n = 3). Soil microbial biomass carbon was comparatively higher in broadleaved forest than in coniferous and mixed forests, although the difference did not reach the significant level (*P* = 0.40, n = 3). This still agreed well with the higher soil temperature in broadleaved forest. The broadleaved forest with the higher soil temperature and soil organic matter could provide much substrate for microbial organism to decompose and release a larger amount of CO_2_-C at both monthly and annual scale in the studied temperate forests. Another reason for the higher accumulative soil respiration in broadleaved forest was the slightly greater activity of soil microbial organism represented by the higher soil microbial biomass carbon content.

Otherwise, the microbial biomass carbon content was 25.52 mg g SOC^−1^ and much lower in broadleaved forest than 32.11 mg g SOC^−1^ and 50.17 mg g SOC^−1^ in mixed and coniferous forests, respectively. The contribution of SMBC to SOC showed a contrary changing trend compared to soil temperature among these three forest types. This would help explain why the forest plot with higher soil temperature released much more CO_2_-C, because the contribution of SMBC to SOC reflected changes in property of natural ecosystems and demonstrated the status of soil carbon accumulation^24^. In essence, increased temperature might accelerate the transformation of soil organic matter into CO_2_-C by soil microbes. A warming experiment indicated that soil microbial metabolic activity was increased 66% by warming, and new and recent fixed carbon were respired 38% larger than under control temperature conditions^25^. More soil labile carbon was perhaps lost through the motivated heterotrophic respiration, which eventually slowed down the accumulation of soil organic carbon. Therefore, this kind of activation of heterotrophic respiration by higher temperature may be the underlying mechanism for maximum temperature accounting for the annual soil respiration in temperate forests of northern China.

## Material and Methods

### Study sites and experiment layout

This study was conducted at the Taiyueshan Long-Term Forest Ecosystem Research Station (36^°^04′ N, 112^°^06′ E; elevation during 600 – 2,600 m a.s.l.), which situates about 190 km southwest of Taiyuan in Shanxi province of China. Experimental site is about 3 km west to the station in a classic catchment named after Xiaoshegou. This area has four distinct seasons, i.e. spring from March to May, summer from June to August, autumn from September to November, and winter from December to February. However, most of precipitation occurs between June and August with a mean annual precipitation of 600 mm, and the mean annual temperature (MAT) is about 11°C, with 26 °C in the warmest period of July and –23°C in the coldest month of January. The hill height at this study region averages 600 m from the baseline at 1200 m a.s.l. The edaphic characteristic of this hill slope belongs to Eutric Cambisols (FAO classification) or Cinnamon soil (Chinese Classification) with a mean soil depth between 30 cm and 50 cm^26^,^27^. The overstory vegetation consists mainly of *Pinus tabuliformis*, *Quercus wutaishanica*, *Betula dahurica*, *Larix gmelinii var. principis-rupprechtii*, *Tilia mongolica*. The understory shrub community mainly consists of *Corylus mandshurica*, *Corylus heterophylla*, *Acer ginnala*, *Lespedeza bicolor*, *Philadelphus incanus*, *Rosa bella*, *Lonicera chrysantha*. Major herbaceous species includes *Carex lanceolata*, *Spodiopogon sibiricus*, *Rubia chinensis*, *Thalictrum petaloideum*, *Melica pappiana*.

Along three ridges within this catchment, three forest types, i.e. coniferous, broadleaved and mixed forests, were investigated to complete this study. Simultaneously, three forest plots at 20 × 20 m were constructed for each forest type. It was needed to be specified that the six plots of broadleaved and mixed forests were ever used in another research of Zhou *et al.*^28^, who mainly focused on basal rates of soil respiration and their determinants. Three coniferous forest plots were originally used to explore the relationship of soil respiration with soil temperature properties, although they had been built and investigated in 2008 and 2009. These nine forest plots distributed along the catchment topography with coniferous forest plots on northfacing slope, broadleaved forest plots on southfacing slope, and mixed forest plots on top slope. Basic characteristics of these three types of forest community were investigated in late August 2009 as described in Zhou *et al.*^28^ (Table 2).

### Soil respiration measurement

Nine soil collars were permanently inserted into the soil at each forest plot, and soil respiration was periodically measured during the growing season in both 2008 and 2009, using a Li – Cor infrared gas analyzer (LI – 8100, Li – Cor Inc., Lincoln, NE, U.S.A.), equipped with a portable chamber. Concurrently, soil temperature at 10 cm depth close to each soil collar was monitored using a thermocouple probe attached to LI-8100 system. The details about these measurements could be seen in Zhou *et al.*^28^, and soil respiration rate at 10 °C (R_10_) and temperature sensitivity (Q_10_, soil respiration change with a proportional change of 10 °C in soil temperature) were calculated for each forest plot via the Ahrrenius function using instantaneous soil respiration rate and the coupled soil temperature^28^.

Soil temperature at 10 cm depth on each forest plot was hourly and automatically logged from September 1^st^ 2012 to August 31^st^ 2013 using a temperature monitor (HOBO U22-001, Onset, U.S.A.), which was buried 10 cm in the top soil through one year period. As R_10_ and Q_10_ were indicated to be two comparatively constant parameters for a specific ecosystem^28^, then we were able to estimate the soil respiration rate at a given hour with the logged temperature complying with the empirical function (1). No change was proposed to happen in soil temperature over an hour, so did the soil respiration rate. Therefore, the accumulative soil respiration on daily, monthly, or annual scale could be calculated following the function (2).
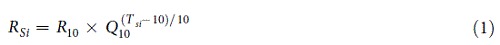

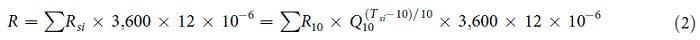
where *R*_*si*_ (μmol CO_2_ m^−2^ s^−1^) represents estimated soil respiration rate at aliquot *i*^*th*^ hour, *T*_*s*_i (°C) is the soil temperature at 10 cm depth automatically logged at aliquot *i*^*th*^ hour, *R*_*10*_ (μmol CO_2_ m^−2^ s^−1^) and *Q*_*10*_ are the calculated parameters of soil respiration using field measurement data of instantaneous soil respiration rate and coupled soil temperature, *R* (g C m^−2^) refers to the accumulative CO_2_ efflux on daily, monthly, or annual scale, 3,600 means 3,600 seconds per hour, 12 means 12 gram carbon per mole CO_2_, and 1 μmol equals 10^−6^ mole. Mean values of *R*_*10*_ and *Q*_*10*_ used in functions (1) and (2) can be seen in Table 1 for each forest type.

In order to explore the effects of soil temperature properties on soil respiration, we calculated the maximum and the minimum soil temperature, i.e. the soil temperature respectively at the warmest and coldest time point during a month or a year, soil temperature range equaled the maximum temperature minus the minimum temperature during each month, and the effective soil temperature equaled mean daily temperature minus 5°C.

### Analyses of soil biochemical properties

Five soil cores were sampled on each forest plot in late August of 2013 using a cylindrical driller of 4 cm in diameter and 20 cm in depth. Sampled soils were put in polyethylene bags and shipped immediately to the laboratory in a box cooled by ice. Prior to nutrient analysis, soils were sieved with a screen of 2 cm, and were evenly separated into two parts after any visible debris was manually picked out. One subsample was air-dried to constant weight and ground to pass through a 0.2 mm sieve before it was used to determine soil organic carbon (SOC) content via the standard Mebius method^29^. Soil total nitrogen content was determined using the Kjeldahl digestion procedure with a Tector Kjeltec system 1026 Distilling unit^30^. Another subsample was stored in refrigerator at 4 °C, and was analyzed for soil microbial biomass carbon (SMBC) through the chloroform fumigation extraction method no longer than seven days since it was collected^31^. Moist soil (equal 15 g oven-dried soil) was extracted with 60 ml of 0.5 M/L K_2_SO_4_ and was shaking for half an hour in a polyethylene bottle with elastic plug. Another 15 g soil (dry weight basis) was firstly fumigated with chloroform in a glass jar with CO_2_-free air, and was incubated for 24 hours at 25 °C in a dark microcosm with air moisture content of 60%. The fumigated soil was extracted in the same way after the incubation was finished. The total amount of microbial biomass carbon was determined by the difference between K_2_SO_4_ – extracted carbon in fumigated and non-fumigated soil using K_ec_ factor of 0.38. Simultaneously, soil bulk density was determined using a cylindrical soil driller of 100 cm^3^ and oven-dried at 110 °C to constant weight in laboratory. Soil pH was measured in deionized H_2_O via Sartorius AG (PB-10, Sartorius, Germany).

### Data analysis

All biophysical variables including soil respiration, soil temperature, and soil resource content, etc. were averaged across forest plots for each forest type when they were analyzed in this article. Analysis of variance (ANOVA) at α = 0.05 significance level was used to test the effect of forest type on biophysical variables. All data analyses were performed using the software of SPSS 15.0. Figures of this paper were completed via the software of Sigmaplot in version 10.0.

## Additional Information

**How to cite this article**: Zhou, Z. *et al.* Maximum temperature accounts for annual soil CO_2_ efflux in temperate forests of Northern China. *Sci. Rep.* **5**, 12142; doi: 10.1038/srep12142 (2015).

## Figures and Tables

**Table 1:** Biophysical and chemical variables of three forest types in northern China.

**Forest type**	**SOC (g/kg)**	**TN (g/kg)**	**C : N**	**SMBC (mg/kg)**	**pH**	**Soil bulk density (g/cm^3^)**	**Litter C stock (g C/m^2^)**	**MAT (℃)**	**R_10_ (μmol CO_2_ m^−2^ s^−1^)**	**Q_10_**
Coniferous forest	18.43 a	1.45 a	12.64 a	828.76 a	6.05 a	1.10 a	649.22 b	6.10 a	1.25 a	2.82 a
Broadleaved forest	33.86 b	2.80 b	11.99 a	868.55 a	6.51 b	0.95 a	359.04 a	7.03 a	1.85 b	3.17 a
Mixed forest	17.38 a	1.47 a	11.75 a	536.59 a	5.76 a	1.04 a	333.78 a	6.41 a	1.62 b	3.36 a

Different lowercase letters in the same column mean significant difference (*P* < 0.05, n = 3) among these three forest types.

**Table 2:** Basic characteristics of the studied temperate forests in northern China.

**Forest type**	**Tree height (m)**	**Diameter at breast height (cm)**	**Plant density (hm**^**−2**^)	**Elevation range (m.a.s.l)**	**Forest age (yr)**	**Slope gradient**
Coniferous forest	10.94	16.50	1,808	1,690	70	17°
Broadleaved forest	7.16	14.35	1492	1,653	70	19°
Mixed forest	7.56	10.41	4033	1,710	70	22°
